# Epigenetic Memory: Lessons From iPS Cells Derived From Human β Cells

**DOI:** 10.3389/fendo.2020.614234

**Published:** 2021-01-19

**Authors:** Shimon Efrat

**Affiliations:** Department of Human Molecular Genetics and Biochemistry, Sackler School of Medicine, Tel Aviv University, Tel Aviv, Israel

**Keywords:** Epigenetic memory, Islet β Cells, pluripotent stem cell differentiation, ATAC-seq, Foxa2****

## Abstract

Incomplete reprogramming of somatic cells into induced pluripotent stem cells (iPSCs) may be responsible for the heterogeneity in differentiation capacity observed among iPSC lines. It remains unclear whether it results from stochastic reprogramming events, or reflects consistent genetic or cell-of-origin differences. Some evidence suggests that epigenetic memory predisposes iPSCs to enhanced differentiation into the parental cell type. We investigated iPSCs reprogrammed from human pancreatic islet β cells (BiPSCs), as a step in development of a robust differentiation protocol for generation of β-like cells. BiPSCs derived from multiple human donors manifested enhanced and reproducible spontaneous and induced differentiation towards insulin-producing cells, compared with iPSCs derived from isogenic non-β-cell types and fibroblast-derived iPSCs (FiPSCs). Genome-wide analyses of open chromatin in BiPSCs and FiPSCs identified thousands of differential open chromatin sites (DOCs) between the two iPSC types. DOCs more open in BiPSCs (Bi-DOCs) were significantly enriched for known regulators of endodermal development, including bivalent and weak enhancers, and FOXA2 binding sites. Bi-DOCs were associated with genes related to pancreas development and β-cell function. These studies provide evidence for reproducible epigenetic memory in BiPSCs. Bi-DOCs may provide clues to genes and pathways involved in the differentiation process, which could be manipulated to increase the efficiency and reproducibility of differentiation of pluripotent stem cells from non-β-cell sources.

## Incomplete Reprogramming Into iPSCs

Reprogramming of somatic cells into induced pluripotent stem cells (iPSCs) using Yamanaka’s combination of four transcription factors (OCT4, SOX2, KLF4, and c-MYC, together termed OSKM) (1) has opened new avenues for in-vitro generation of multiple human differentiated cell types for disease modeling, drug screening, and regenerative medicine. Initially iPSCs derived from multiple cell types have been expected to manifest similar differentiation capacities, resembling those of human embryonic stem cells (ESCs). However, accumulated experience has shown a great heterogeneity in differentiation capacity among iPSC lines. This heterogeneity is thought to result from incomplete reprogramming.

The mechanisms activated by ectopic expression of OSKM in somatic cells, which result in reprogramming to pluripotency, are only partly understood. This prolonged process, lasting several weeks, involves multiple events, including silencing of somatic cell genes and activation of pluripotency-associated genes. The low efficiency of OSKM-mediated reprogramming is thought to reflect the stochastic nature of these complex events. Only a small fraction of cells acquire pluripotency, as judged by accepted assays (e.g. differentiation into cells from the three embryonic germ layers in embryoid bodies (EB), and teratoma formation assay). OSKM factors function by binding to chromatin regions and inducing their remodeling, thereby activating or repressing gene expression. Evidence suggests that broad epigenetic changes are among early key events of the reprogramming process (2).

The epigenetic landscape, shaped by DNA methylation and histone modifications, is critical for maintaining cell identity. Erasing cell-specific patterns of epigenetic modifications, and replacing them with pluripotency patterns, are central to cell reprogramming to pluripotency. Ample evidence supports the incomplete and varying erasure of the original epigenetic marks in both mouse and human iPSC lines. However, it remains unclear whether these variations represent consistent patterns, based on the cell type of origin, or genetic differences among donors, or reflect stochastic differences caused by low efficiency of the reprogramming mechanisms (**Table 1**). While epigenetic memory may not necessarily affect gene expression patterns in iPSCs (3), likely due to missing transcription factors, it is expected to affect differentiation capacity towards specific cell fates. This may limit some applications of iPSCs, but at the same time may predispose iPSCs to enhanced differentiation into the parental cell type, thereby facilitating generation of cells for specific uses.

**Table 1:** Possible sources of iPSC variability.

Possible source of iPSC variability	Expected phenotype
Stochastic reprogramming events	Differences among clones from a single cell type of origin from a single donor
Donor-related	Similarity among clones from multiple cell types of origin from a single donor; differences among iPSCs from multiple donors
Cell type of origin	Similarity among iPSCs from the same cell type of origin from multiple donors

## iPSC Differences Related to the Cell Type of Origin

### Mouse iPSCS

Early comparison of mouse iPSCs derived from bone marrow progenitor cells, dermal fibroblasts, and neural progenitor cells, identified residual DNA methylation signatures characteristic of the somatic tissue of origin (4). These differences favored iPSC differentiation towards the donor cell type, while restricting alternative cell fates. These results were contrasted to the methylation and differentiation patterns of nuclear-transfer-derived pluripotent stem cells, which were more similar to those of ESCs. Similar findings were reported in early-passage iPSCs obtained from mouse fibroblasts, hematopoietic and myogenic cells, which exhibited distinct epigenetic patterns (5). These patterns were reflected in different transcriptional profiles of the iPSCs, and in their differentiation efficiency into embryoid bodies (EBs) and hematopoietic cell types. These differences were eroded with iPSC passaging, suggesting that epigenetic memory was a transient phenomenon. iPSCs derived from mouse neonatal cardiomyocytes (CMs) were also shown to differentiate toward CMs more efficiently than fibroblast-derived iPSCs (FiPSCs) or mouse ESCs (6).

### Human iPSCs

A number of reports documented similar epigenetic memory in human iPSCs. Kim et al. compared DNA methylation profiles and differentiation potential of iPSCs derived from human umbilical cord blood and neonatal keratinocytes (7). They identified distinct genome-wide DNA methylation patterns in iPSCs derived from each cell type, resulting from both incomplete erasure of tissue-specific methylation and aberrant de-novo methylation. These differences did not disappear upon extended passaging. Ohi et al. observed that human iPSCs generated from hepatocytes, skin fibroblasts, and melanocytes, retained some transcriptional characteristics of the original cells at low passages, which could be partially explained by incomplete promoter DNA methylation (8). They noticed that incompletely silenced genes tended to be isolated from other genes that were repressed during reprogramming, indicating that silencing of isolated genes may be less efficient. Global DNA methylation analyses of iPSCs reprogrammed from human cornea limbal epithelial stem cells (LESC) showed gene methylation patterns similar to those of the parental cells, and different from those of FiPSCs (9). Upon differentiation, LESC-derived iPSCs expressed higher levels of LESC markers, compared with FiPSCs.

## iPSC Differences Related to Donors and Stochastic Variability

In contrast to these findings, which associated epigenetic and differentiation differences among iPSCs with their cell type of origin, other studies supported a donor-related or stochastic basis for these differences. In one such study (10), whole-genome profiling of DNA methylation in five human iPSC lines derived from adipocytes and fibroblasts (including 3 subclones of a single line) identified over 1,000 differentially methylated sites, most of them associated with CG islands and genes, indicating stochastic interclone reprogramming variability. Two other studies (11, 12) surveyed 16–18 iPSC lines each, derived from fibroblasts and peripheral blood cells of four human donors in each study. Both studies concluded that the majority of transcriptional and DNA methylome differences among iPSCs, as well as differences in their differentiation capacity towards the hematopoietic cell lineage, could be attributed to the donor, rather than the cell type of origin, indicating that genetic differences among donors can result in reproducible reprogramming differences. Finally, two studies, which analyzed differences in gene expression patterns in human iPSCs, concluded that genetic differences between individual donors were the major cause of transcriptional variation between lines. One of these studies (13) compared 25 iPSC lines from four donors and three tissues. The second study compared two human ESC lines with genetically matched iPSCs derived from fibroblasts differentiated from each ESC line (14). Their findings showed reproducible gene expression patterns among each ESC line and three iPSC clones derived from it, and variations compared with the other ESC line and its iPSC derivatives. The study concluded that ESCs and iPSCs exhibited similar gene expression patterns, and that the donor genetic background was responsible for transcriptional variations among pluripotent stem cell lines.

The donor genetic background may also influence the differentiation capacity of ESC lines. However, in contrast to the relatively large numbers of iPSC lines that have been generated by multiple laboratories, data on differentiation of ESC lines accumulated to date is based on a small number of established ESC lines commonly used by all research groups. This limited number, which is a result of ethical barriers to generation of new ESC lines, does not allow proper comparisons of variations among ESC lines in differentiation potential into specific cell lineages.

## Epigenetic Memory in iPSCs Derived From Human β Cells

Directed in-vitro differentiation of iPSCs into β-like cells is a promising approach for generation of abundant insulin-producing cells for cell therapy of diabetes, disease modeling and drug screening. Despite significant progress (15–21), current differentiation protocols result in cells with heterogeneous and immature phenotype, and suffer from low efficiency and variability among iPSC lines. In a step towards developing a more robust differentiation protocol, we investigated iPSCs reprogrammed from human pancreatic islet β cells (BiPSCs). This approach depended on lineage tracing of human β-cell-derived (BCD) cells within the heterogeneous cell population in cultures of isolated human islets (22). The stable genetic label allowed positive identification of the β-cell origin of individual iPSC clones, that otherwise would be difficult to distinguish from iPSCs derived from non-β-cell types present in the expanded islet cell culture. Initial studies of four BiPSC lines derived from β cells of three nondiabetic human donors established their pluripotency, as judged by standard assays (23). Nevertheless, chromatin immunoprecipitation (ChIP) analyses showed that the levels of histone H3 acetylation, a hallmark of open chromatin structure, at the *INS* and *PDX1* loci in BiPSCs were similar to those found in human islets and BCD cells, and significantly higher than those in FiPSCs, as well as in two iPSC lines derived from isogenic islet non-β cells (termed PiPSCs) from two of the donors. BiPSCs also exhibited significantly lower DNA methylation levels, characteristic of transcribed genes, in genes expressed in human islet cells, such as *PDX1*, compares with FiPSCs. Despite the open chromatin marks, β-cell genes were not expressed in BCD and BiPS cells.

The epigenetic memory of BiPSCs was associated with higher expression levels of *PDX1*, *FOXA2*, and *INS* transcripts following spontaneous differentiation into EBs and teratomas, compared with those derived from FiPSCs and PiPSCs, and an enhanced induced differentiation capacity into insulin-producing cells in mice transplanted with BiPSC-derived endocrine progenitors, following the protocol of Kroon et al. (24). The epigenetic phenotype and differentiation capacity of BiPSCs were reproducible among the four lines obtained from three human donors, and appeared stable within the passage range analyzed (passages 10–20).

To identify genes and pathways, which may be responsible for the enhanced and reproducible differentiation capacity of BiPSCs, we performed a global analysis of chromatin sites differentially open in BiPSCs, compared with FiPSCs, using an Assay for Transposase Accessible Chromatin with high-throughput sequencing (ATAC-seq) (25). For this analysis we generated five new BiPSC lines from three nondiabetic donors, which were compared to five FiPSC lines from two nondiabetic donors. All these lines passed pluripotency assays, and EBs generated from BiPSCs at passages 9-12 showed enhanced spontaneous expression of *FOXA2*, *PDX1*, and *INS*, compared with those derived from FiPSCs (25), similarly to the four BiPSC lines in the initial study.

Despite high overall similarity in open chromatin between the two iPSC types, the ATAC-seq analysis identified thousands of significantly differential open chromatin (DOC) sites between BiPSCs and FiPSCs, most of which were more open in BiPSCs (Bi-DOCs). Bi-DOCS overlapped gene regulatory elements known to be involved in development, such as weak enhancers (marked by H3K4me1) and bivalent enhancers and promoters (marked by H3K27me3), especially near genes involved in endodermal development, such as *FOXA2* and its target genes, and pancreas development, such as *PDX1*, and *NKX2-2*, as well as genes expressed in mature β cells, such as *INS*. The ATAC-seq data for these four genes (*FOXA2*, *PDX1*, *NKX2-2*, and *INS*) was validated by H3K4me3 ChIP analysis, which found higher levels of this open chromatin mark in the promoter regions of all four genes in BiPSCs, compares with FiPSCs. These findings could explain the enhanced expression of these genes in EBs generated from BiPSCs, compared with those derived from FiPSCs.

The relevance of Bi-DOCs to differentiation towards islet cells was analyzed by comparing directed differentiation of BiPSCs and FiPSCs into definitive endoderm (DE) and pancreatic progenitor (PP) cells according to the protocol of Rezania et al. (16). Global transcriptome analyses by RNA-seq identified 567 protein-coding genes expressed at higher levels in BiPSC-derived DE, compared with FiPSC-derived DE, and 181 genes expressed at higher levels in BiPSC-derived PP, compared with FiPSC-derived PP (25). These genes were significantly enriched for genes mapping near Bi-DOCs. Among genes expressed at higher levels in BiPSC at both stages, the most prominent was estrogen receptor 1 (*ESR1*; 3.5-fold and 53.4-fold higher in DE and PP, respectively) and several of its target genes. 17β-estradiol (E2) has been shown to protect mouse β cells from apoptosis by signaling through estrogen receptor (ER) α encoded by *ESR1* (26). ERα activity has been reported to increase *Neurog3* expression and β-cell proliferation in a mouse model of pancreas partial duct ligation, and during mouse islet development (27). It has been suggested to regulate endocrine lineage specification through downregulation of NOTCH signaling. Thus, inclusion of E2 in the culture medium at key stages of the in-vitro differentiation protocol may increase its efficiency and reproducibility.

## Conclusion

Overall, the analyses of Bi-DOCs support the existence of reproducible epigenetic memory in BiPSCs. The association between Bi-DOCs and gene expression levels at early stages of the in-vitro differentiation protocol provides a plausible explanation for the enhanced differentiation capacity of BiPSCs into the β-cell lineage, compared with pluripotent stem cells from a non-β-cell source. Both chromatin structure and differentiation capacity were reproducible in a combined number of nine BiPSC lines from six human islet donors in two separate studies. Bi-DOCs may provide clues to genes and pathways involved in the differentiation process, which could be manipulated to increase the efficiency and reproducibility of differentiation of pluripotent stem cells from other sources. Such manipulations could include activation of candidate genes using CRISPR-on approaches or small-molecule compounds.

## Author Contributions

The author confirms being the sole contributor of this work and has approved it for publication.

## Conflict of Interest

The author declares that the research was conducted in the absence of any commercial or financial relationships that could be construed as a potential conflict of interest.
